# Application of a Novel Lytic Podoviridae Phage Pu20 for Biological Control of Drug-Resistant *Salmonella* in Liquid Eggs

**DOI:** 10.3390/pathogens10010034

**Published:** 2021-01-04

**Authors:** Yu Zhang, Yifeng Ding, Wanning Li, Wenjuan Zhu, Jia Wang, Xiaohong Wang

**Affiliations:** 1 Key Laboratory of Environment Correlative Dietology, Huazhong Agricultural University, Wuhan 430070, China; 123zy@webmail.hzau.edu.cn (Y.Z.); yifengding@webmail.hzau.edu.cn (Y.D.); wangjia@mail.hzau.edu.cn (J.W.); 2 College of Food Science and Technology, Huazhong Agricultural University, Wuhan 430070, China; wanning.li@wondfo.com.cn (W.L.); zhuwenjuan@webmail.hzau.edu.cn (W.Z.)

**Keywords:** bacteriophage, *Salmonella*, biocontrol, antibacterial activity, genomic analysis

## Abstract

*Salmonella* is a globally distributed zoonotic pathogen. Among them, *S. pullorum* is a host-specific pathogen that seriously affects the development of the poultry breeding industry in China. It mainly infects chickens and can cause white scabs, and the mortality rate after infection is almost 100%. As antibiotics are widely used in animal feed and other production processes, *Salmonella* resistance has gradually increased. Therefore, there is an increasing need to develop new technologies to control multi-drug resistant (MDR) pathogens and confirm their actual effectiveness in the target food matrix. Bacteriophage can efficiently and specifically lyse bacteria, and will be a potential bactericide to replace antibiotics. In this study, 34 strains of *Salmonella* bacteriophages were isolated from environmental resources. Therein, phage Pu20 with the widest host spectrum had the strongest ability to lyse tested *Salmonella* strains. Further studies showed that Pu20 had high pH tolerance and heat resistance, short incubation period. Pu20 can effectively inhibit the growth of two strains of MDR *Salmonella* in liquid egg white and yolk at 4 °C and 25 °C, respectively. According to morphological and phylogenetic analysis, Pu20 belongs to the Podoviridae family. Genomic analysis of Pu20 indicates a linear 59435 bp dsDNA sequence with no homology to virulence or antibiotic resistance-related genes. Together, these results sheds light on the potential biocontrol application value of Pu20 in food products.

## 1. Introduction

*Salmonella* is a common zoonotic pathogen that has a global distribution. It is likely to cause infectious diseases such as gastroenteritis, sepsis, and typhoid fever, posing a huge threat to human and animal health. More than 2600 serotypes of *Salmonella* have been found all over the world now. Only certain serotypes of *Salmonella* can infect humans and animals [1]. Among them, *S. pullorum* is a host-specific pathogen that seriously affects the development of the poultry industry in China, it has a wide range, high pathogenicity and mainly infects chickens [2]. It can cause white diarrhea, and the mortality of chickens after infection is almost 100%. This disease not only brings serious harm to the chicken industry, causing huge economic losses, but also expands the spread of *Salmonella* in poultry through excrement and eggs. It can be colonized in the intestine of poultry. When processing carcasses, it contaminates chicken meat and then enters the human food chain, becoming a potential source of human *Salmonella* infection [3]. According to the Centers for Disease Control and Prevention (CDC), approximately 1 million cases of *Salmonella* infection occur in the United States each year, and 200 million to 1.3 billion cases occur worldwide [4]. In the United States, data provided by the Foodborne Active Surveillance Network (FoodNet) shows that *Salmonella* infections are most commonly reported in the region, with an incidence rate of 17.6 cases per million people per year [5]. In addition, 70% to 80% of bacterial food poisoning in China is due to consumption of foods contaminated with *Salmonella* [6]. More than 90% of these foods are livestock and poultry products, and 252 cases of *Salmonella* food poisoning occurred between 1998 and 2002 of the incidents, 90 were caused by eating eggs and egg products [7].

In recent decades, as antibiotics are widely used in animal feed and other production processes, the resistance of *Salmonella* has gradually increased, and even super bacteria have been produced, especially those resistant to β-lactam and fluoroquinolones [8,9,10]. The threat of *Salmonella* to human and animal health is also increasing [11]. At least 2 million people in the United States are infected with antibiotic-resistant bacteria every year, and 23,000 people die as a result. Compared with direct infection with antibiotic-resistant bacteria, its complications can cause more deaths. The survey data shows that the multi-drug resistance (MDR) of *Salmonella* has increased from 20% to 30% in the 1990s to 70% in the beginning of this century, and the *Salmonella* drug resistance rate will continue to increase significantly over time [12]. Selectivity of antibacterial drugs will become narrower and narrower, which becomes a major problem leading to foodborne diseases [13].

As a potential antibacterial agent, bacteriophage has gradually attracted researchers' attention because of its safety, high efficiency, and specificity [14,15]. Bacteriophages are widely distributed in nature and can be isolated from a variety of different sources, such as poultry feces, saliva, and soil and sewage [16]. Bacteriophage is a virus that specifically breaks down bacteria [17]. It is also an important part of the human microbiome [18]. It has the characteristics of high efficiency, high specificity, easy to prepare in large quantities. It is colorless, tasteless, and does not affect the flavor of the food itself. It is a relatively safe and efficient fungicide [19,20].

The number of bacteriophages isolated from the environment is increasing, and many of them have been tested to control *Salmonella* in various food vectors, such as milk, meat, fruit, and vegetables [21,22,23]. Due to the accelerated emergence of drug-resistant strains, it is still necessary to find more broad-spectrum phages with potential for resistance to MDR *Salmonella* [24]. At the same time, it is essential to test the feasibility of these phages for biocontrol of MDR *Salmonella* in high-risk foods such as eggs, because the food matrix may affect the performance of phages [25,26,27]. The purpose of this research is to establish a phage-based biological control strategy against multidrug-resistant *Salmonella* and test its antibacterial effect in eggs. Taking *Salmonella pullorum* as host bacteria, 34 strains of *Salmonella pullorum* phages were isolated and purified from sewage and food samples. Among them, phage Pu20 was selected for further study because of its broad host spectrum. The biological characteristics such as morphology, adsorption, one-step growth curve, heat resistance, and pH value of phage Pu20 were determined. Then, the effect of Pu20 on the growth of MDR *Salmonella* in medium and liquid eggs was further studied. To evaluate its genetic safety, sequencing and molecular analysis of Pu20 genome were done. Our research shows that broad-spectrum phage Pu20 belonging to the Podoviridae family, which has the potential as a promising antibacterial agent against MDR *Salmonella* in food products.

## 2. Materials and Methods

### 2.1. Bacterial Strains and Culture Conditions

Detailed information on bacterial strains used in this study is listed in Table S1. They were either collected from American Type Culture Collection (ATCC), National Center for Medical Culture Collections (CMCC; Beijing, China), the China Center of Industrial Culture Collection (CICC), the National Collection of Type Cultures (NCTC), China Veterinary Culture Collection Center (CVCC), and Shanghai Jiao Tong University (SJTU), or isolated from environmental sources. All 49 strains were stored frozen at −80 °C in 20% (vol/vol) glycerol and cultured in LB medium at 37 °C.

### 2.2. Enrichment, Isolation and Purification of Salmonella pullorum Bacteriophages

A total of 10 sewage samples (sewer sewage, domestic sewage, and rainwater isolated from different areas of Wuhan city) and 10 chicken meat samples (chicken and chicken blood are also isolated from farmers’ markets and supermarkets in different regions of Wuhan city) were used for phages separation. Water samples were centrifuged (Allegra X-30R Centrifuge, Beckman Coulter, Shanghai, China) at 10,000× *g* for 10 min at 37 °C, followed by filtration through a 0.22 μm filter (Millipore, Ireland) [28]. 

Chicken meat samples were homogenized in samples buffer (2 g/L MgSO_4_·7H_2_O, 5.8 g/L NaCl, and 0.05 L of l mol/L Tris-HCl; pH 7.5) with 10 times dilution. Then, 10 mL of homogenate was mixed with 10 mL of Luria–Bertani (LB) broth and incubated at 37 °C for 18 h with shaking. Then, 2.5% chloroform was added and incubated at room temperature for 5 min. Centrifuge for 15 min at 8000× *g*, and then, the supernatant was filtered with a 0.22 μm filter to obtain filtered samples [29].

*Salmonella pullorum* CVCC534 and CVCC519 were used to isolate phages from filtered samples. The filtered sample was mixed with the sterilized LB broth (1.0 g of peptone, 0.5 g of yeast extract, and 1.0 g of NaCl in 0.1 L of distilled water; pH 7.3) and suspensions of host *Salmonella* strains. The mixtures were incubated at 37 °C with shaking (160 rpm) for 12–18 h. After incubation, the mixed samples were centrifuged at 8000× *g* for 15 min and filtered with 0.22 μm filters. Then, 10 μL of filtered samples were seeded onto a double-layer agar plate (LB with 1.5% agar as the bottom layer, LB with 0.7% agar mixed with a suspension of host strain as the overlay) and incubated at 37 °C for 16–24 h. Samples exhibited a clear zone were considered as positive. For purification, independent, large, smooth-edged plaques were selected and mixed with 100 μL host bacteria in 1 mL LB broth and incubated at 37 °C for 12–18 h, centrifuge at 8000 r/min for 10 min at 4 °C and filter the bacteria with a 0.22 μm filter. Then the filters were purified using double-layer agar plate again. The purification was performed 3–4 times and purified phages were stored in 20% glycerol at −80 °C.

### 2.3. Host Range Study

Bacterial strains used for the host range study are listed in Table S1. The lytic ability of the isolated phages against different strains was verified by spot test [30]. Suspensions of tested strains (100 μL) were mixed with LB containing 0.7% agar (3.5 mL), serving as the overlay. LB containing 1.5% agar (15 mL) was served as the bottom layer. Phage lysates (5 μL) were spotted onto a double-layer agar plate containing the lawns of target strains and incubated at 37 °C for 18–24 h. The host range of the phages was assessed using a validated scoring method by evaluating the characteristics of plaques. Then phages with a wide range of lysis were tested for their ability to lyse 10 multi-drug resistant *Salmonella* strains.

### 2.4. Morphological Observation by TEM

Phage lysates were ultra-centrifuged (Optima^TM^ XE-100 Ultracentrifuge, Beckman Coulter) at 40,000 rpm/min for 1 h at 4 °C and resuspended in 0.1 mol/L ammonium acetate. Phosphotungstic acid (PTA) negative staining method was used for the transmission electron microscopy (TEM) observation. The copper grid for TEM was immersed into the phage suspension for 10 min and then stained by PTA solution (volume fraction of 2%, pH 7) for 10 min [31]. The morphology of phage Pu20 was determined by TEM (Hitachi H-7000FA, Tokyo, Japan) and analyzed by the software Digital Micrograph Demo 3.9.1.

### 2.5. Optimal Multiplicity of Infection

According to the ratio of multiple infections of 0.001, 0.01, 0.1, 1, 10, 100, and 1000, *S. pullorum* CVCC534 (100 μL) and Pu20 phage (100 μL) mixed with LB broth (800 μL) were incubated at 37 °C with shaking (100 rpm) for 3.5 h. After incubation, the mixtures were centrifuged at 11,000× *g* for 10 min. The double-layer agar plate method was used to determine the phage titer [32]. The multiplicity of infection with the highest titer is the optimal multiplicity of infection (MOI) of this phage. Phage titer (plaque forming units (PFU/mL)) = dilution gradient × dilution factor × 10.

### 2.6. Adsorption Rate

The lysate (5 mL) of phage Pu20 was mixed with an equal volume of the suspension of *S. pullorum* CVCC534 (Multiplicity of infection (MOI) = 0.1). The mixture was then incubated at 37 °C for 50 min with shaking at 160 rpm/min. Every 5 min, 300 μL of the mixture was taken and placed on ice for 30 s. Then the mixture was centrifuged at 7000 rpm/min for 30 s and the supernatant was diluted and spotted onto a double-layer agar plate containing the lawns of host strains to determine the phage titer. Adsorption rate (%) = (initial phage titer − phage titer after incubation)/initial phage titer [23].

### 2.7. One-Step Growth Curve

Burst sizes and latent periods of phage Pu20 were determined by the one-step growth curve as previously described [33,34]. The lysate (500 μL) of phage Pu20 was mixed with an equal volume of the suspension of *S. pullorum* CVCC534 (MOI = 0.1). Then the mixture was incubated at 37 °C for 20 min with shaking at 160 rpm/min. After incubation, the mixture was centrifuged at 7000× *g* for 2 min. The pellet was washed twice with LB broth and resuspended with 10 mL of preheated LB. Then the suspension was immediately incubated at 37 °C for 3 h with shaking at 160 rpm/min. Every 10 min, 300 μL of the sample was taken and centrifuged at 7000 rpm/min for 30 s. The supernatant was diluted and spotted onto a double-layer agar plate containing the lawns of host strains to determine the phage titer. Relative burst size = (final phage titer – initial phage titer)/initial phage titer.

### 2.8. Stability of Phage Pu20 at Different Temperatures and pH

For thermal stability, the lysate of phage Pu20 (1 mL, 10^7^ PFU/mL) was incubated at different temperatures from 30 to 80 °C for 30 min or 60 min. After incubation, the phage suspension was cooled to room temperature and spotted on a double-layer agar plate to determine the phage titer. For the stability at different pH, lysate of phage Pu20 (100 μL, 10^8^ PFU/mL) was added into 900 μL of LB at different pH (2–13). The mixture was incubated at 37 °C for 2 h. After incubation, the phage suspension was diluted at the end of the reaction time and spotted on a double-layer agar plate to determine the phage titer.

### 2.9. Pu20 Inhibited the Growth of MDR Salmonella enterica Serovar Enteritidis and Typhimurium

From ten multi-drug resistant *Salmonella* strains tested above, two *Salmonella enterica* serovar Enteritidis 11561 and *Salmonella enterica* serovar Typhimurium SJTUF 13277 were selected to test the inhibitory ability of phage Pu29. Respectively, 100 μL two bacterial suspensions (10^5^ CFU/mL) were mixed with 100 μL of phage Pu20 lysate (10^3^–10^8^ PFU/mL). Then the mixtures were incubated at 37 °C for 12 h. The lytic capacity was depicted by measuring the OD_600_ at a 1-hour interval. One hundred μL of suspension (10^5^ CFU/mL) of host strains mixed with an equal volume of LB was served as a positive control, whereas 100 μL of the phage lysate (10^7^ PFU/mL) mixed with an equal volume of LB was served as a negative control.

### 2.10. Biocontrol of Salmonella enterica Serovar Enteritidis and Salmonella enterica Serovar Typhimurium in Liquid Eggs by Pu20

Eggs were rinsed with distilled water and 75% ethanol and then sterilized by UV light for 30 min. Egg yolk and egg white were spotted onto LB agar plate and incubated at 37 °C for the confirmation of sterility. One hundred μL of lysate of phage Pu20 (10^8^or 10^9^ PFU/mL) was added into 9.8 mL of sterile egg yolk or egg white that was inoculated with 100 μL of suspension (10^5^ CFU/mL, in phosphate buffered saline (PBS)) of *S. enteritidis* 11561 or *S. typhimurium* SJTUF 13277. An equal volume of PBS was added into control groups. The mixtures were incubated at 4 °C or 25 °C, respectively. Samples were collected at 0, 1, 3, 6, 12, and 24 h post-incubation and recoverable bacteria from liquid eggs were determined by serial plating methods [35,36].

### 2.11. Structural Protein Analysis of Pu20

With reference to the method of extraction of lambda phage particles in the Third Edition of the Molecular Cloning Experiment Guide, phage Pu20 particles were concentrated. The highly purified phage sample was subjected to sodium dodecyl sulfate polyacrylamide gel electrophoresis (SDS-PAGE) using 12% acrylamide concentration. Coomassie brilliant blue staining solution was used to stain the samples. Samples were stained on a circular shaking shaker at room temperature for about 3 h. Then discard the staining solution, add decolorizing solution to decolorize the gel, and later capture the image with a gel scanner (Bio-rad Gel Doc XR+, Shanghai, China).

### 2.12. Analysis of Pu20 Genome

The DNA extraction of Pu20 genome was performed using previously validated phage genomic DNA extraction method [30]. The DNA concentration was measured by Qubit fluorometer (Thermo Fisher, Waltham, Massachusetts, USA) and DNA quality was proved by agarose gel electrophoresis. Purified DNA samples were stored at −20 °C for further use. Phage genome was sequenced on the Illumina HiSeq platform (Illumina, San Diego, CA, USA) with 2 × 150 bp paired-end runs and assembled using the software MicrobeTrakr plus 0.9.1. Protein encoding genes were predicted by the software Prodigal 2.6.0 [37]. Annotation was performed using MyRast (My-Rast, https://rast.nmpdr.org/) and manually checked using BLASTP (BLASTP, https://blast.ncbi.nlm.nih.gov/Blast.cgi) and Uniprot (Uniprot, https://www.uniprot.org/) [38,39,40]. Genes encoding tRNAs were screened by tRNAScan-SE (tRNAScan-SE, http://lowelab.ucsc.edu/tRNAscan-SE/) [41]. Putative virulence factors were screened by the Virulence Factor Database and antibiotic resistance genes were screened by the Comprehensive Antibiotic Resistance Database [42]. Comparative circular genome map of phage genomes was depicted by the software BRIG Comparison Tool [43]. According to the classification of viruses by ICTV (The International Committee on Taxonomy of Viruses) and BLASTn (BLASTn, https://blast.ncbi.nlm.nih.gov/Blast.cgi) of the NCBI database, phage with higher similarity was selected [44,45]. The phylogenetic tree was constructed based on the protein sequence of terminase large subunit using the software MEGA 7 with the Neighbor-Joining method and 500 bootstraps [46]. Sequence alignment was performed by ClustalW 2.1 and tree visualization was performed with ITOL (https://itol.embl.de/itol.cgi).

### 2.13. Statistical Analysis

Data analysis was performed using Prism 6.0 (GraphPad Software, La Jolla, CA, USA). All experiments were conducted in triplicate except the application of phage Pu20 in liquid eggs, which was performed in duplicates. The one way ANOVA with Tukey’s multiple-comparison was used to determine the significance among groups at a significance level of *p* < 0.05.

## 3. Results and Discussions

### 3.1. Isolation of Phages

In this study, a total of 10 sewage samples and 10 chicken samples were collected. Among them, 23 phages were isolated using *Salmonella pullorum* CVCC519 as the host bacteria, and 11 phages were isolated using *Salmonella pullorum* CVCC534 as the host bacteria (Table 1). 

### 3.2. Phage Pu20 Exhibited the Broadest Spectrum Against Salmonella

**Figure 1:**
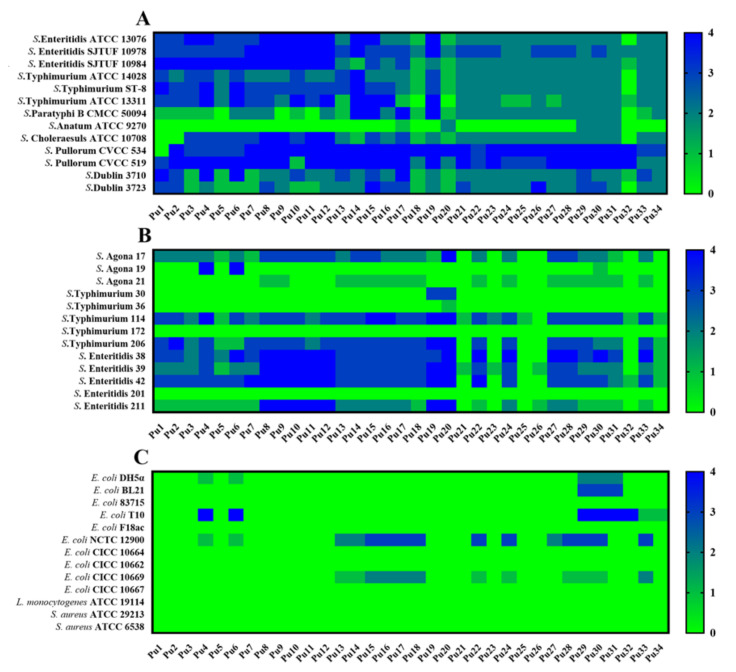
Host spectrum of isolated 34 phages. (**A**) Host spectrum of isolated phages against 13 preserved non-resistant *Salmonella* strains of seven serotypes. (**B**) Host spectrum of isolated phages against 13 preserved drug-resistant *Salmonella* strains of three serotypes. (**C**) Host spectrum of isolated phages against 13 preserved bacterial strains from other genera. Lytic capability is indicated by heat maps. Numbers from 0 to 4 are corresponding to colors from green to blue; “4” indicates a completely clear plaque; “3” indicates a generally clear plaque with the faint hazy background; “2” indicates obvious turbidity throughout clear lytic zone; “1” indicates an individually opaque plaque; “0” indicates no lytic zone.

Among 34 isolated phages, Pu20 infected 21 of the 26 tested *Salmonella* strains to varying degrees, including 9 resistant *Salmonella* strains to varying degrees. In contrast, Pu20 cannot lyse strains of other genera, such as *Escherichia coli*, *Listeria monocytogenes* and *Staphylococcus aureus* (Figure 1). Furthermore, Pu20 effectively infected and lysed multidrug-resistant (MDR) *Salmonella* strains that were isolated from food and clinical samples (Table 2). In view of these results, phage Pu20 was selected for further study.

### 3.3. Morphology of Pu20

**Figure 2:**
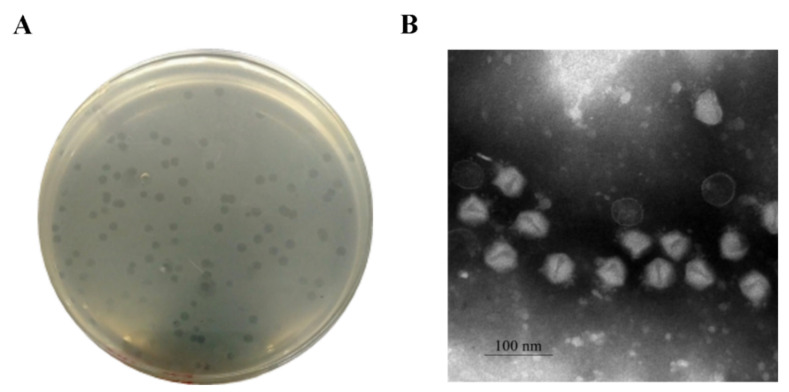
Morphological characteristics of phage Pu20. (**A**) Bacteriophage plaques of Pu20. (**B**) Morphology of phage Pu20 presented by TEM. The bar indicates the magnification size of 100 nm.

Pu20 could form larger and clear plaque with a diameter of 3.5–4.0 mm in double-layer agar plate (Figure 2A). Morphology of Pu20 was depicted by TEM (Figure 2B). It revealed that the head of Pu20 is stereo-symmetric and is a typical icosahedron structure with a head diameter of 42.23 nm. The tail length of Pu20 is approximately 18.66 nm. Morphologic analysis pointed out Pu20 belongs to the Podoviridae family [47]. 

### 3.4. Optimal Multiplicity of Infection

**Figure 3:**
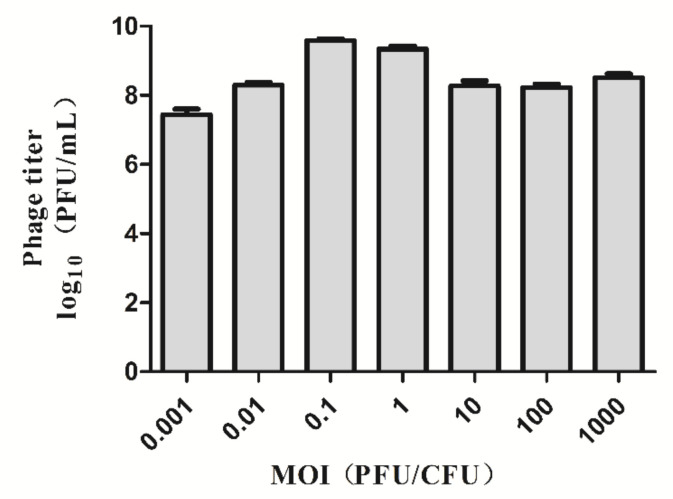
Determination of optimal multiplicity of infection (MOI) of phage Pu20.

The optimal multiplicity of infection results of phage Pu20 are shown in Figure 3. It is known that the phage Pu20 has the highest titer at MOI = 0.1; therefore, the optimal multiplicity of infection of phage Pu20 is 0.1, indicating that a large number of host bacteria can be lysed with a small number of phage.

### 3.5. Adsorption Rate

**Figure 4:**
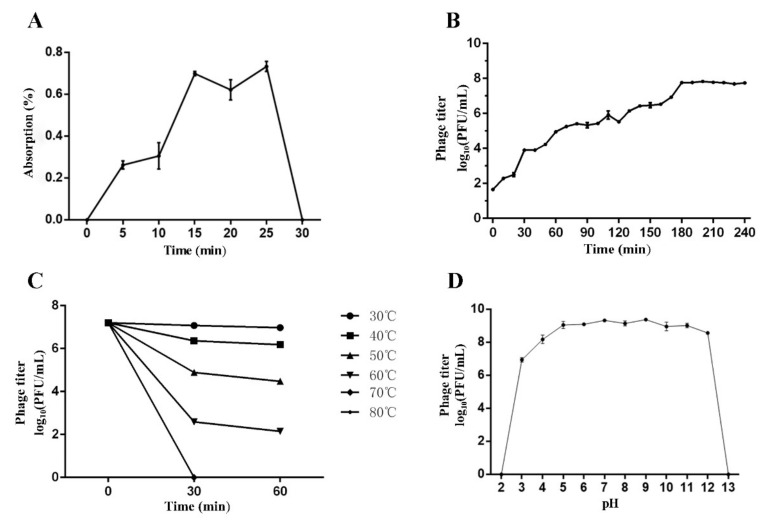
Biological characterization of Phage Pu20. (**A**) Adsorption rate. (**B**) One-step growth curve. (**C**) Stability of Pu20 at different temperatures. (**D**) Stability of Pu20 at different pH.

The adsorption rate of phage Pu20 showed an upward trend from 0 to 25 min, and reached a peak after reaching 25 min, 73.30%. After 25 min, the adsorption rate fell sharply (Figure 4A).

### 3.6. One-Step Growth Curve

The incubation period of phage Pu20 at MOI of 0.1 is 20 min. The lysis period of Pu20 was 180 min. After a short incubation period, Pu20 showed an exponential increase from 20 min to 180 min. The burst size of Pu20 was calculated to be approximately 34 PFU/cell (Figure 4B). Compared with other bacteriophages reported in the literature, Pu20 had a shorter incubation period, which may be a reflection of high lytic activity [21,29,48]. Generally, phages with short incubation periods can lyse more bacterial cells in a certain period of time and were therefore more suitable for biological control [49,50].

### 3.7. Stability

The initial titer of *Salmonella* bacteriophage Pu20 was 1.7 × 10^7^ PFU/mL. The titer remained stable at 30 °C without significant change. At 40 °C to 60 °C, the phage titer decreased sharply with increasing temperature. The titer decreased sharply at 0 min–30 min, the rate of decline slowed down from 30 min to 60 min, and the phage was completely inactivated after 30 min at 70 °C (Figure 4C). Pu20 showed moderate thermal stability, this result was similar to the temperature tolerance of several bacteriophages previously reported, and they all lost activity at 70 °C [50,51]. Pasteurization was the most common method for pretreatment of liquid egg products, usually at 60 °C for a few minutes, while Pu20 had certain activity at 60 °C, indicating that the idea of using bacteriophages as an auxiliary heat treatment to kill pathogens may be feasible.

The phage Pu20 can maintain high activity at pH 3 to 12, and the fluctuation of phage activity was small. In contrast, Pu20 was significantly affected at pH < 3 and pH > 12. When the pH was 2 or 13, the activity of the phage decreases to almost zero (Figure 4D). Pu20 had a stable titer at a pH of 3–12 and showed a high pH tolerance. Therefore, the stability under acidic and alkaline conditions allowed phages to be used in food substrates with different pH values. For example, fruits and yogurt, which usually had a low pH value, and milk which pH was neutral. In addition, eggs were relatively special [52]. The whole egg was close to pH neutral. However, egg white was one of the few foods that were naturally alkaline, and its pH during storage was between 7.6 and 9.2. In contrast, the pH of the egg yolk during storage was between 6.0 and 6.9. The large pH changes and inherent composition of eggs pose a challenge for the use of bacteriophages in this matrix. Obviously, Pu20, which had higher pH tolerance, did not conflict with these matrices [53].

### 3.8. Lytic Ability of Pu20 on MDR Salmonella Strains

**Figure 5:**
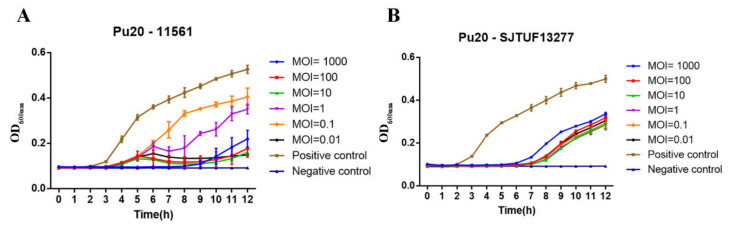
Lytic ability of Pu20 against *Salmonella enteritidis* 11561(**A**) and *Salmonella typhimurium* SJTUF13277 (**B**).

We then tested whether Pu20 could inhibit the dynamic growth of MDR *Salmonella*. Without Pu20 treatment, the two strains reached exponential growth phase at 3 h after inoculation and increased sharply from 3 to 12 h (Figure 5A,B). With Pu20 treatment at MOI of 1000 and 100, a significant rise of *S. enteritidis* 11561 was observed after 4 h. Except for other MOIs of 1000, upward trend of *S. enteritidis* 11561 effectively blocked by Pu20 from 6 to 8 h, the blocking rebounded within 8 to 12 h after phage Pu20 treatment (Figure 5A). Correspondingly, on all tested MOIs, the growth of *S. typhimurium* SJTUF 13277 was continuously inhibited for 6 h after Pu20 treatment, and then a significant rebound was observed (Figure 5B). Pathogens could develop resistance to bacteriophages, which hinders the widespread use of bacteriophages. Our previous studies had shown that within five hours of combined culture, the emergence of phage resistance could be observed in *Salmonella*, as evidenced by the disappearance/weakened inhibition of *Salmonella* growth [35]. 

### 3.9. Biocontrol of Salmonella enterica Serovar Enteritidis in Liquid Eggs

**Figure 6:**
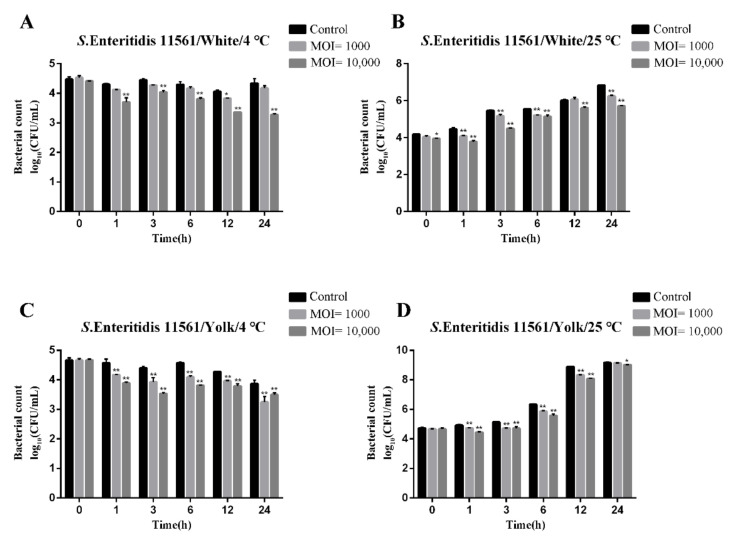
Application of phage Pu20 for the biocontrol of *S. enteritidis* 11561 in liquid eggs. (**A**) Biocontrol of *S. enteritidis* 11561 in egg white at 4 °C. (**B**) Biocontrol of *S. enteritidis* 11561 in egg white at 25 °C. (**C**) Biocontrol of *S. enteritidis* 11561 in egg yolk at 4 °C. (**D**) Biocontrol of *S. enteritidis* 11561 in egg yolk at 25 °C, * Significant; ** Highly significant, *p*-value < 0.05.

The phage Pu20 had a significant antibacterial effect on *Salmonella enteritidis* 11561 in egg white at 4 °C and 25 °C when MOI = 10,000 (*p* < 0.01). Compared with the control group, the number of viable bacteria in the test group decreased by up to 1.06 log_10_ CFU/mL and 1.12 log_10_ CFU/mL at 4 °C and 25 °C for 24 h, respectively, and the maximum antibacterial efficiency was 91.30% and 92.40%, respectively. when MOI = 1000 (*p* < 0.05), 25 °C Significant antibacterial effect (*p* < 0.05), but no significant antibacterial effect at 4 °C (Figure 6A,B). When MOI = 1000, after 12 h of treatment at 4 °C, compared with the control group, the number of viable bacteria in the test group was reduced by 0.23 log_10_ CFU/mL, and the antibacterial efficiency was the highest at 40.62%, after 24 h of treatment at 25 °C, Pu20 had the highest antibacterial efficiency against *Salmonella enteritidis* 11561 in egg white, reaching 73.90%.

The phage Pu20 had a significant antibacterial effect on *Salmonella enteritidis* 11561 in egg yolk at 4 °C and 25 °C (*p* < 0.01), at 4 °C, MOI = 10,000 had significant antibacterial effect when treated for 3 h (*p* < 0.05). Compared with the control group, the number of viable bacteria in the experimental group decreased by 0.87 log_10_ CFU/mL, and the antibacterial rate was 86.38%. However, there is no obvious antibacterial effect under the conditions of MOI = 1000 and 25 °C for 24 h (Figure 6C,D). The bacteriostatic effect of the 4 °C test group was better than that of the 25 °C group, which might be because the low temperature can inhibit the growth of *Salmonella*, and the growth rate of *Salmonella* at 25 °C is faster than the speed of phage lytic bacteria. 

### 3.10. Biocontrol of Salmonella enterica Serovar Typhimurium in Liquid Eggs

**Figure 7:**
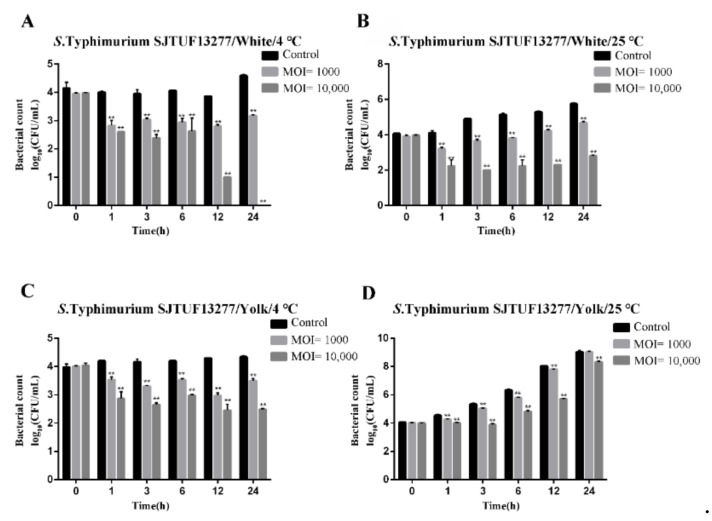
Application of phage Pu20 for the biocontrol of *S. typhimurium* SJTUF 13277 in liquid eggs. (**A**) Biocontrol of *S. typhimurium* SJTUF 13277 in egg white at 4 °C. (**B**) Biocontrol of *S. typhimurium* SJTUF 13277 in egg white at 25 °C. (**C**) Biocontrol of *S. typhimurium* SJTUF 13277 in egg yolk at 4 °C. (**D**) Biocontrol of *S*.Typhimurium SJTUF 13277 in egg yolk at 25 °C; ** Highly significant, *p*-value < 0.05.

The phage Pu20 had an extremely significant antibacterial effect on *Salmonella typhimurium* SJTUF13277 in egg white at 4 °C and 25 °C (*p* < 0.01) (Figure 7A,B). The number of viable bacteria in the control group was basically maintained near the initial concentration. Even when the MOI was 10000 and treated at 4 °C for 24h, the number of viable bacteria in the test group decreased by 4.60 log_10_ CFU/mL compared with the control group, antibacterial efficiency reached 100.00%. 

The phage Pu20 had a significant antibacterial effect on *Salmonella typhimurium* SJTUF13277 in egg yolk at 4 °C and 25 °C (*p* < 0.01), but there was no significant antibacterial effect when treated at 25 °C and MOI = 1000 for 24 h. (*p* < 0.05) (Figure 7C,D). When MOI = 10,000 and at 25 °C for 12 h, the antibacterial efficiency of Pu20 in egg yolk was the highest, up to 99.51%. The antibacterial effect of the egg white group was better than that of the egg yolk group. The analysis might be due to the fact that egg white contains lysozyme, egg transferrin, and other bacteriostatic substances, which reduced the number of live bacteria in the egg white. Under certain conditions, the growth of phage will reduce the bacteriostatic effect of phage. Under what conditions can the bacteriostatic substance in egg white cooperate with phage to inhibit bacteria, these were the issues that need to be further investigated in the application [53].

### 3.11. Structural Protein

**Figure 8:**
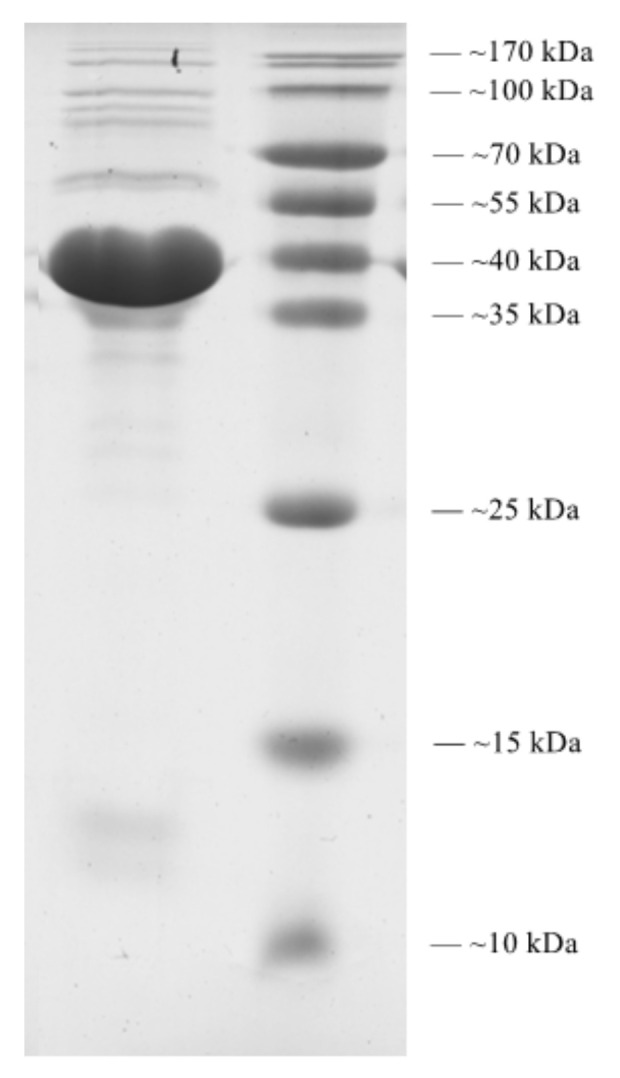
SDS-PAGE of phage Pu20 structural protein. The left lane is a 15 μL protein sample, and the right lane is a 5 μL medium molecular weight protein standard marker.

In order to analyze the structural proteins of Pu20, purified phage particles were separated by SDS-PAGE. At least seven distinct protein bands, with molecular weights ranging from 37 to 170 kDa, were visualized in the SDS-PAGE gel (Figure 8). The band with the largest content was analyzed by Quantity One software with a molecular weight of about 37 kDa, and the corresponding protein had the largest copy number in the phage Pu20 particles. This most important structural protein is most likely the phage capsid protein, but it is finally determined that each structural protein needs to be identified by mass spectrometry or complete genome annotation.

### 3.12. Overview of the Pu20 Genome

**Figure 9:**
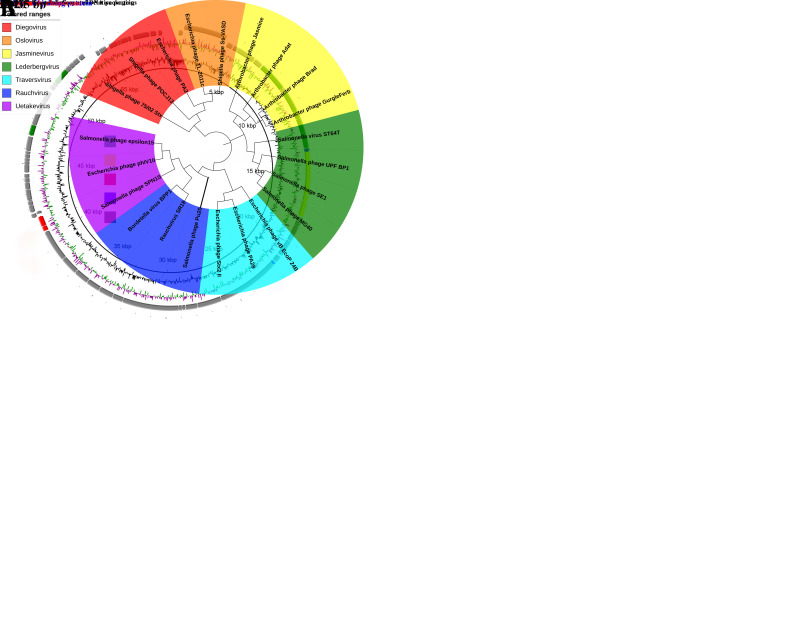
Genome function analysis and phylogenetic analysis of Pu20. (**A**) A comparative circular genome map generated using the BRIG comparison tool. The meaning of each circle (from the inside): (1) the gene scale of bp; (2) GC content; (3) GC skew, values greater than zero are in green and the smaller are in magenta; (4) genomic gene annotation. (**B**) Phylogenetic analysis of Pu20 large terminase subunit (thick solid line). The ClustalW program was used to align the large terminase subunits, and a phylogenetic tree was generated using the nearest neighbor ligation method with 1000 repetitions of the bootstrap program. Different types of phages are represented by different colors.

In order to better understand Pu20 at the molecular level, we analyzed the entire genome of Pu20 and found that its sequence length was 59435bp and GC content was 56.26%. Using tRNAscan-SE to predict tRNA genes in the whole genome, the results showed no tRNA. A total of 74 open reading frames (ORFs) were identified in the Pu20 genome (Table S2). Among them, 15 ORFs are predicted to encode functional proteins, while 59 ORFs are predicted to encode hypothetical proteins and proteins with unknown functions. Among the 15 functional genes, 7 genes are involved in nucleic acid metabolism and DNA packaging, 4 genes are structural protein genes other than the tail, 2 genes are proteins related to the tail, and 2 cleavage module encoding genes (Figure 9A). Using Res Finder to predict the absence of antibiotic resistance genes in the bacteriophage Pu20 genome, and using Virulence Finder to predict the absence of virulence genes in the phage Pu20 genome.

According to the classification of Podoviridae family recorded by ICTV, 21 phages with higher scores were selected from the results of BLASTn comparison of Pu20 genome sequence in the NCBI database (Table S3). Among them, *Rauchvirus* SR18 and *Bordetella* phage BPP-1 have the highest similarities with phage Pu20, 79.31%, and 70.69% respectively. The large terminase subunit is a relatively conserved gene in phage. In the process of phage assembly, it is responsible for cleaving this subunit into tandem DNA to form mature linear DNA, and then the phage displays different ends. Phages with similar amino acid sequences of terminase subunits usually exhibit similar mechanisms in DNA packaging [54]. Phylogenetic analysis showed that Pu20 clustered into the Rauchvirus genus group, indicating that the phage Pu20 may belong to a new species of Rauchvirus in the Podoviridae family (Figure 9B).

## 4. Conclusions

In summary, this study proposes a broad-spectrum *Salmonella* phage Pu20 isolated from sewage, which has a strong lytic effect on MDR *Salmonella* strains. It has high pH tolerance and heat resistance, short incubation period. Pu20 significantly inhibited the growth of MDR *Salmonella* in high-risk liquid eggs at different temperatures. Morphological and genomic analysis revealed that Pu20 belongs to the Podoviridae family Bacteriophage. No virulence and anti-biocide related genes were found in the Pu20 genome, suggesting that Pu20 is a candidate gene for MDR *Salmonella* biocontrol in high-risk foods.

## Figures and Tables

**Table 1:** Source information of 34 isolated *Salmonella* phages.

Host	Total	Source Information	
		Sewage	Chicken
CVCC519	23	Pu1,Pu2,Pu3,Pu4,Pu5,Pu6,Pu7,Pu8,Pu16,Pu24,Pu27,Pu28,Pu29,Pu30	Pu9,Pu10,Pu11,Pu12,Pu13,Pu14,Pu15,Pu17,Pu31
CVCC534	11	Pu18,Pu19,Pu20, Pu25,Pu26,Pu33,Pu34	Pu21,Pu22,Pu23,Pu32

**Table 2:** The lytic ability of phage Pu20 against 10 multidrug-resistant *Salmonella* strains.

**Bacteriophage**	***Salmonella* Indiana**		***Salmonella enteritidis***		***Salmonella typhimurium***					
	13500	13520	10960	11561	10855	SJTUF 13306	SJTUF 13277	SJTUF 13336	SJTUF 13337	SJTUF 13350
Pu20	++++	+++	+++	++++	++	++	++++	+++	++	++

“++++,” completely clear; “+++,” clearing throughout, but with faint hazy background; “++,” substantial turbidity throughout the cleared zone.

## Data Availability

The data presented in this study are contained within the article or supplementary materials.

## Supplementary Materials

The following are available online at https://www.mdpi.com/2076-0817/10/1/34/s1, Table S1: Bacterial strains used in this study. Table S2: Pu20 genome annotation. Table S3: Podoviridae bacteriophage phages similar to Pu20 genome.
