# Supplementary material for: Application of a Novel Lytic Podoviridae Phage Pu20 for Biological Control of Drug-Resistant Salmonella in Liquid Eggs
Source: Pathogens. 2021 Jan 4;10(1):34. doi: 10.3390/pathogens10010034 (PMC7823707; doi:10.3390/pathogens10010034)
Supplement: Supplementary file 1 [file pathogens-10-00034-s001.zip › Supplementary Table S3 - resubmit 2.docx]

**Supplementary Table S3.** *Podoviridae* bacteriophage phages similar to Pu20 genome

| Phage name | Family | Subfamily | Genus | Genome  length（kb） | | Country | Accession | Reference |
| --- | --- | --- | --- | --- | --- | --- | --- | --- |
| *Rauchvirus* SR18 | Podoviridae | unknow | Rauchvirus | 45.260 | USA | | MN746332.1 | unpublished |
| *Bordetella* phage BPP-1 | Podoviridae | unknow | Rauchvirus | 42.493 | USA | | NC_005357.1 | [55] |
| *Escherichia* phage PA2 | Podoviridae | Sepvirinae | Oslovirus | 63.569 | USA | | NC_028449.1 | unpublished |
| *Escherichia* phage  TL-2011c | Podoviridae | Sepvirinae | Oslovirus | 60.532 | Norway | | NC_019442.1 | unpublished |
| *Shigella* phage Ss-VASD | Podoviridae | Sepvirinae | Oslovirus | 62.851 | USA | | NC_028685.1 | unpublished |
| *Escherichia* virus 24B | Podoviridae | Sepvirinae | Traversvirus | 57.677 | UK | | NC_027984.1 | unpublished |
| *Escherichia* phage PA28 | Podoviridae | Sepvirinae | Traversvirus | 61.292 | USA | | NC_041935.1 | unpublished |
| *Escherichia* virus Stx2 II | Podoviridae | Sepvirinae | Traversvirus | 62.706 | Japan | | NC_004914.3 | [56] |
| *Arthrobacter* phage Adat | Podoviridae | unknow | Jasminevirus | 45.428 | USA | | NC_042020.1 | unpublished |
| *Arthrobacter* phage Jasmine | Podoviridae | unknow | Jasminevirus | 46.723 | USA | | NC_041875.1 | unpublished |
| *Arthrobacter* phage Brad | Podoviridae | unknow | Jasminevirus | 45.418 | USA | | MH450114.1 | unpublished |
| *Arthrobacter* phage GurgleFerb | Podoviridae | unknow | Jasminevirus | 45.426 | USA | | MF668273.1 | unpublished |
| *Salmonella* phage SE1 | Podoviridae | unknow | Lederbergvirus | 41.941 | Spain | | NC_011802.1 | [57] |
| *Enterobacteria* phage ST64T | Podoviridae | unknow | Lederbergvirus | 40.679 | Australia | | NC_004348.1 | unpublished |
| *Salmonella* phage UPF_BP1 | Podoviridae | unknow | Lederbergvirus | 39.902 | Brazil | | NC_047875.1 | [58] |
| *Salmonella* phage MG40 | Podoviridae | unknow | Lederbergvirus | 40.315 | USA | | MT774487.1 | [59] |
| *Shigella* phage 75/02 Stx | Podoviridae | Sepvirinae | Diegovirus | 60.875 | Hungary | | NC_029120.1 | unpublished |
| *Shigella* phage POCJ13 | Podoviridae | Sepvirinae | Diegovirus | 62.699 | USA | | NC_025434.1 | unpublished |
| *Escherichia* phage phiV10 | Podoviridae | unknow | Uetakevirus | 39.104 | USA | | NC_007804.2 | [60] |
| *Enterobacteria* phage epsilon15 | Podoviridae | unknow | Uetakevirus | 39.672 | USA | | NC_004775.2 | [61] |
| *Salmonella* phage SPN1S | Podoviridae | unknow | Uetakevirus | 38.684 | Korea | | NC_016761.1 | [62] |

References

1. Dai, W.; Hodes, A.; Hui, W.H.; Gingery, M.; Miller, J.F.; Zhou, Z.H. Three-Dimensional Structure of Tropism-Switching Bordetella Bacteriophage. *Natl. Acad. Sci.* **2010**, *107*, 4347–4352, doi:10.1073/pnas.0915008107.
2. Sato, T.; Shimizu, T.; Watarai, M.; Kobayashi, M.; Kano, S.; Hamabata, T.; Takeda, Y.; Yamasaki, S. Genome Analysis of a Novel Shiga Toxin 1 (Stx1)-Converting Phage Which Is Closely Related to Stx2-Converting Phages but Not to Other Stx1-Converting Phages. *J. Bacteriol.* **2003**, *185*, 3966–3971, doi:10.1128/jb.185.13.3966-3971.2003.
3. Martí, N.B.i. Caracterització Del Genoma I anàlisi Del Regulador Gènic Del Bacteriòfag SE1 de *Salmonella* Enterica. *Universitat Autònoma De Barcelona*. **2006**, 11, 46–53.
4. Rizzo, N.N.; Pottker, E.S.; Webber, B.; Borges, K.A.; Duarte, S.C.; Levandowski, R.; Dos Santos, L.R.; Rodrigues, L.B. Effect of Two Lytic Bacteriophages against Multidrug-Resistant and Biofilm-Forming Salmonella Gallinarum from Poultry. *Br. Poult. Sci.* **2020**, 1–6, doi:10.1080/00071668.2020.1805724.
5. Gilcrease, E.B.; Leavitt, J.C.; Casjens, S. Genome Sequence of Salmonella Enterica Serovar Typhimurium Bacteriophage MG40. *Microbiol. Resour. Announc.* **2020**, *9*, 9, doi:10.1128/mra.00905-20.
6. Kim, J.; Kim, M.; Kim, S.; Ryu, S. Sensitive Detection of Viable Escherichia coli O157:H7 from Foods Using a Luciferase-Reporter Phage PhiV10lux. *Int. J. Food Microbiol.* **2017**, *254*, 11–17, doi:10.1016/j.ijfoodmicro.2017.05.002.
7. Jiang, W.; Chang, J.; Jakana, J.; Weigele, P.; King, J.; Chiu, W. Structure of Epsilon15 Bacteriophage Reveals Genome Organization and DNA Packaging/Injection Apparatus. *Nat. Cell Biol.* **2006**, *439*, 612–616, doi:10.1038/nature04487.
8. Park, Y.; Lim, J.-A.; Kong, M.; Ryu, S.; Rhee, S. Structure of Bacteriophage SPN1S Endolysin Reveals an Unusual Two-Module Fold for the Peptidoglycan Lytic and Binding Activity. *Mol. Microbiol.* **2014**, *92*, 316–325, doi:10.1111/mmi.12555.
