# Supplementary material for: Application of a Novel Lytic Podoviridae Phage Pu20 for Biological Control of Drug-Resistant Salmonella in Liquid Eggs
Source: Pathogens. 2021 Jan 4;10(1):34. doi: 10.3390/pathogens10010034 (PMC7823707; doi:10.3390/pathogens10010034)
Supplement: Supplementary file 1 [file pathogens-10-00034-s001.zip › Supplementary Table S1 - resubmit 2.docx]

**Supplementary Table S1.** Bacterial strains used in this study.

| **Name** | **Description** | **Reference** |
| --- | --- | --- |
| ATCC13076 | *Salmonella enterica* serovar Enteritidis, standard strain | Laboratory stock |
| SJTUF10978 | *Salmonella enterica* serovar Enteritidis, isolate | Laboratory stock |
| SJTUF10984 | *Salmonella enterica* serovar Enteritidis, isolate | Laboratory stock |
| ATCC14028 | *Salmonella enterica* serovar Typhimurium, standard strain | Laboratory stock |
| ATCC13311 | *Salmonella enterica* serovar Typhimurium, standard strain | Laboratory stock |
| ST-8 | *Salmonella enterica* serovar Typhimurium, isolate | Laboratory stock |
| CMCC50094 | *Salmonella enterica* serovar Paratyphi B, standard strain | Laboratory stock |
| ATCC9270 | *Salmonella enterica* serovar Anatum, standard strain | Laboratory stock |
| ATCC10708 | *Salmonella enterica* serovar Choleraesuls, standard strain | Laboratory stock |
| CVCC534 | *Salmonella enterica* serovar Pullorum, standard strain | Laboratory stock |
| CVCC519 | *Salmonella enterica* serovar Pullorum, isolate | Laboratory stock |
| 3710 | *Salmonella enterica* serovar Dublin, isolate | Laboratory stock |
| 3723 | *Salmonella enterica* serovar Dublin, isolate | Laboratory stock |
| DH5α | *Escherichia coli*, engineered strain | Laboratory stock |
| BL21 | *Escherichia coli*, engineered strain | Laboratory stock |
| 83715 | *Escherichia coli*, isolate | Laboratory stock |
| T10 | *Escherichia coli*, engineered strain | Laboratory stock |
| F18ac | *Escherichia coli*, isolate | Laboratory stock |
| NCTC12900 | *Escherichia coli* O157:H7, standard strain | Laboratory stock |
| CICC10664 | *Escherichia coli* (EPEC), standard strain | Laboratory stock |
| CICC10662 | *Escherichia coli* (EIEC), standard strain | Laboratory stock |
| CICC10669 | *Escherichia coli* (STEC), standard strain | Laboratory stock |
| CICC10667 | *Escherichia coli* (ETEC), standard strain | Laboratory stock |
| ATCC19114 | *Listeria monocytogenes*, standard strain | Laboratory stock |
| ATCC29213 | *Staphylococcus aureus*, standard strain | Laboratory stock |
| ATCC6538 | *Staphylococcus aureus*, standard strain | Laboratory stock |
| 17 | *Salmonella enterica* serovar Agona, isolate, multidrug-resistant | Laboratory stock |
| 19 | *Salmonella enterica* serovar Agona, isolate, multidrug-resistant | Laboratory stock |
| 21 | *Salmonella enterica* serovar Agona, isolate, chloramphenicol-resistant | Laboratory stock |
| 30 | *Salmonella enterica* serovar Typhimurium, isolate, multidrug-resistant | Laboratory stock |
| 36 | *Salmonella enterica* serovar Typhimurium, isolate, multidrug-resistant | Laboratory stock |
| 114 | *Salmonella enterica* serovar Typhimurium, isolate, multidrug-resistant | Laboratory stock |
| 172 | *Salmonella enterica* serovar Typhimurium, isolate, multidrug-resistant | Laboratory stock |
| 206 | *Salmonella enterica* serovar Typhimurium, isolate, multidrug-resistant | Laboratory stock |
| 10855 | *Salmonella enterica* serovar Typhimurium, isolate, multidrug-resistant | Laboratory stock |
| SJTUF13306 | *Salmonella enterica* serovar Typhimurium, isolate, multidrug-resistant | Laboratory stock |
| SJTUF13277 | *Salmonella enterica* serovar Typhimurium, isolate, multidrug-resistant^2^ | Laboratory stock |
| SJTUF13336 | *Salmonella enterica* serovar Typhimurium, isolate, multidrug-resistant | Laboratory stock |
| SJTUF13337 | *Salmonella enterica* serovar Typhimurium, isolate, multidrug-resistant | Laboratory stock |
| SJTUF13350 | *Salmonella enterica* serovar Typhimurium, isolate, multidrug-resistant | Laboratory stock |
| 38 | *Salmonella enterica* serovar Enteritidis, isolate, multidrug-resistant | Laboratory stock |
| 39 | *Salmonella enterica* serovar Enteritidis, isolate, multidrug-resistant | Laboratory stock |
| 42 | *Salmonella enterica* serovar Enteritidis, isolate, nalidixic acid-resistant | Laboratory stock |
| 201 | *Salmonella enterica* serovar Enteritidis, isolate, multidrug-resistant | Laboratory stock |
| 211 | *Salmonella enterica* serovar Enteritidis, isolate, multidrug-resistant | Laboratory stock |
| 10960 | *Salmonella enterica* serovar Enteritidis, isolate, multidrug-resistant | Laboratory stock |
| 11561 | *Salmonella enterica* serovar Enteritidis, isolate, multidrug-resistant^1^ | Laboratory stock |
| 13500 | *Salmonella enterica* serovar Indiana, isolate, multidrug-resistant | Laboratory stock |
| 13520 | *Salmonella enterica* serovar Indiana, isolate, multidrug-resistant | Laboratory stock |

^1^*S*. Enteritidis 11561 was resistant to ampicillin, azithromycin, ceftriaxone, fosfomycin, nalidixic acid, streptomycin, sulfamethoxazole, and trimethoprim-sulphamethoxazole.

^2^*S*. Typhimurium SJTUF 13277 was resistant to ampicillin, chloramphenicol, ciprofloxacin, gentamicin, kanamycin, nalidixic acid, ofloxacin, streptomycin, sulfamethoxazole, trimethoprim-sulphamethoxazole, and tetracyclin
