# Supplementary material for: Application of a Novel Lytic Podoviridae Phage Pu20 for Biological Control of Drug-Resistant Salmonella in Liquid Eggs
Source: Pathogens. 2021 Jan 4;10(1):34. doi: 10.3390/pathogens10010034 (PMC7823707; doi:10.3390/pathogens10010034)
Supplement: Supplementary file 1 [file pathogens-10-00034-s001.zip › Supplementary Table S2 - resubmit 2.docx]

**Supplementary Table S2**. Pu20 genome annotation.

| ORF | Strand | Gene positions | Functions |
| --- | --- | --- | --- |
| 1 | + | 570-863 | hypothetical protein |
| 2 | - | 902-3484 | hypothetical protein |
| 3 | - | 3481-3762 | hypothetical protein |
| 4 | + | 4002-4421 | hypothetical protein |
| 5 | + | 4483-4812 | hypothetical protein |
| 6 | + | 4805-6142 | hypothetical protein |
| 7 | + | 6197-6793 | DUF2815 family protein |
| 8 | + | 6860-8899 | DNA polymerase I |
| 9 | + | 8901-9188 | VRR-NUC domain-containing protein |
| 10 | + | 9181-10710 | DNA helicase, phage-associated |
| 11 | + | 10697-11266 | terminase small subunit |
| 12 | + | 11256-13331 | terminase large subunit |
| 13 | + | 13342-13596 | head-to-tail joining protein W |
| 14 | + | 13593-15272 | phage portal protein |
| 15 | + | 15269-16585 | prohead protease ClpP/capsid assembly protease |
| 16 | + | 16600-16995 | head decoration protein |
| 17 | + | 17008-18072 | major capsid protein |
| 18 | + | 18134-18427 | hypothetical protein |
| 19 | + | 18430-18795 | hypothetical protein |
| 20 | + | 18795-19421 | hypothetical protein |
| 21 | + | 19418-19921 | hypothetical protein |
| 22 | + | 19935-21080 | hypothetical protein |
| 23 | + | 21177-21638 | hypothetical protein |
| 24 | + | 21683-21880 | tail assembly chaperone |
| 25 | + | 21873-26168 | hypothetical protein |
| 26 | + | 26174-27862 | hypothetical protein |
| 27 | + | 27872-28690 | DUF2163 domain-containing protein |
| 28 | + | 28702-28932 | hypothetical protein |
| 29 | + | 28932-29171 | hypothetical protein |
| 30 | + | 29161-33051 | hypothetical protein |
| 31 | + | 33051-33791 | hypothetical protein |
| 32 | + | 33801-34808 | hypothetical protein |
| 33 | + | 34819-35781 | hypothetical protein |
| 34 | + | 35795-36814 | hypothetical protein |
| 35 | + | 36829-38058 | hypothetical protein |
| 36 | + | 38068-40263 | hypothetical protein |
| 37 | + | 40326-40664 | endolysin 2 |
| 38 | + | 40668-41381 | endolysin 1 |
| 39 | - | 41387-41668 | hypothetical protein |
| 40 | + | 41559-41762 | hypothetical protein |

| 41 | - | 41763-42224 | hypothetical protein |
| --- | --- | --- | --- |
| 42 | - | 42221-42505 | hypothetical protein |
| 43 | - | 42502-42591 | NinC protein |
| 44 | - | 42581-43294 | hypothetical protein |
| 45 | - | 43296-43748 | hypothetical protein |
| 46 | - | 43745-44056 | hypothetical protein |
| 47 | - | 44053-44334 | hypothetical protein |
| 48 | - | 44415-44927 | hypothetical protein |
| 49 | - | 44909-45163 | hypothetical protein |
| 50 | - | 45153-45368 | hypothetical protein |
| 51 | - | 45365-45634 | hypothetical protein |
| 52 | - | 45631-45825 | hypothetical protein |
| 53 | - | 45836-46810 | hypothetical protein |
| 54 | - | 46888-47592 | N-6-adenine-methyltransferase |
| 55 | - | 47592-48692 | hypothetical protein |
| 56 | - | 48689-49279 | hypothetical protein |
| 57 | - | 49276-50022 | hypothetical protein |
| 58 | - | 50212-50487 | hypothetical protein |
| 59 | - | 50492-50725 | hypothetical protein |
| 60 | - | 50728-51804 | recombination-associated protein RdgC |
| 61 | - | 51785-52126 | hypothetical protein |
| 62 | - | 52113-52448 | hypothetical protein |
| 63 | - | 52435-52875 | hypothetical protein |
| 64 | - | 52947-53318 | hypothetical protein |
| 65 | + | 54079-54336 | hypothetical protein |
| 66 | + | 54355-54894 | hypothetical protein |
| 67 | + | 55112-55735 | hypothetical protein |
| 68 | + | 55747-55950 | hypothetical protein |
| 69 | + | 55961-56509 | hypothetical protein |
| 70 | + | 56506-56727 | hypothetical protein |
| 71 | + | 56853-58007 | hypothetical protein |
| 72 | + | 58060-58266 | hypothetical protein |
| 73 | + | 58163-58483 | hypothetical protein |
| 74 | + | 58612-58884 | hypothetical protein |
